# The relevance of BDNF for neuroprotection and neuroplasticity in multiple sclerosis

**DOI:** 10.3389/fneur.2024.1385042

**Published:** 2024-08-01

**Authors:** Michelle Maiworm

**Affiliations:** Department of Neurology, University Hospital Frankfurt, Frankfurt, Germany

**Keywords:** BDNF, brain-derived neurotrophic factor, multiple sclerosis, neuroplasticity, neuroprotection, remyelination, neuronal damage

## Abstract

**Background:**

Neuroplasticity as a mechanism to overcome central nervous system injury resulting from different neurological diseases has gained increasing attention in recent years. However, deficiency of these repair mechanisms leads to the accumulation of neuronal damage and therefore long-term disability. To date, the mechanisms by which remyelination occurs and why the extent of remyelination differs interindividually between multiple sclerosis patients regardless of the disease course are unclear. A member of the neurotrophins family, the brain-derived neurotrophic factor (BDNF) has received particular attention in this context as it is thought to play a central role in remyelination and thus neuroplasticity, neuroprotection, and memory.

**Objective:**

To analyse the current literature regarding BDNF in different areas of multiple sclerosis and to provide an overview of the current state of knowledge in this field.

**Conclusion:**

To date, studies assessing the role of BDNF in patients with multiple sclerosis remain inconclusive. However, there is emerging evidence for a beneficial effect of BDNF in multiple sclerosis, as studies reporting positive effects on clinical as well as MRI characteristics outweighed studies assuming detrimental effects of BDNF. Furthermore, studies regarding the Val66Met polymorphism have not conclusively determined whether this is a protective or harmful factor in multiple sclerosis, but again most studies hypothesized a protective effect through modulation of BDNF secretion and anti-inflammatory effects with different effects in healthy controls and patients with multiple sclerosis, possibly due to the pro-inflammatory milieu in patients with multiple sclerosis. Further studies with larger cohorts and longitudinal follow-ups are needed to improve our understanding of the effects of BDNF in the central nervous system, especially in the context of multiple sclerosis.

## 1 Introduction

Neuroplasticity as a mechanism to overcome central nervous system (CNS) injury has gained increasing attention in the context of different neurological diseases during the last years. In the field of multiple sclerosis (MS), histopathological studies have shown that remyelination can be found in a subset of patients with inflammatory processes as well as marked neurodegeneration (1). However, deficiency of these repair mechanisms leads to accumulation of neuronal damage and therefore long-term disability (2).

To date, the mechanisms by which remyelination occurs and why the extent of remyelination differs interindividually between MS patients regardless of the disease course are unclear. However, as increasingly effective therapeutic options designed to halt inflammation and slow neurodegeneration are available, approaches that might reverse preexisting, already accumulated damage are becoming increasingly important.

### 1.1 General information on BDNF and its cellular effects

A member of the neurotrophins family, the brain-derived neurotrophic factor (BDNF) has received particular attention in this context. As the most abundant growth factor in the brain, BDNF is thought to be of uttermost importance for the regulation of neuronal survival and death, neuronal proliferation as well as differentiation, synaptic plasticity, and repair processes, thus playing a key role in neuroprotection and neuroplasticity (3–6).

In the CNS, BDNF is synthesized and secreted by neurons as well as activated astrocytes and microglial cells. Astrocytes are the first cells to react to inflammatory CNS damage by expressing various surface markers, neurotrophic factors, and cytokines (7), thereby attracting microglia and macrophages to the site of damage. In turn, their activation leads to further release of cytokines as well as neurotrophic factors and phagocytosis of damaged cells (8). Microglia and macrophages exist in three different phenotypes. M0 is the resting state of microglial cells, while M1 and M2 are active forms, with M1 exerting inflammatory and M2 anti-inflammatory effects (9, 10). The M2-polarization can be induced by BDNF via activation of the tropomyosin-related kinase B (TrkB) receptor, which in turn induces the synthesis of anti-inflammatory cytokines and neurotrophic factors such as BDNF (10–12).

BDNF is synthesized and released in an activity-dependent manner (8) and through the expression of TrkB receptors, macrophages and microglia are able to react to BDNF, thus BDNF release occurs in an autocrine manner (8). Therefore, damage of astrocytes exerts neuroprotective effects through recruitment of macrophages and microglia by inducing their BDNF synthesis. This is further supported, as lesions with ongoing inflammation show higher concentrations of BDNF with upregulation of TrkB receptors on damaged neurons. Therefore, signaling of infiltrating microglia and macrophages through BDNF to damaged neurons supports the resolution of inflammation, neuronal survival and regeneration (13).

In the periphery, BDNF is also synthesized and released by platelets, monocytes and macrophages as well as activated T- and B-cells (14–16). Furthermore, endothelial cells in the periphery and in the brain are also capable of secreting BDNF (17–19), which can be triggered by various stimuli such as TNF-α, a proinflammatory cytokine (20) thought to play a pivotal role in MS pathophysiology (21). As endothelial cells release BDNF and also express TrkB receptors, an autocrine effect is hypothesized. However, TNF-α can not only induce the release of BDNF from endothelial cells, but also activates microglia, thus TNF-α can induce further cytokine release, thus amplifying the neuroinflammatory process (22). Yet, besides these deleterious effects, TNF-α also exhibits neuroprotective effects, as it is capable of inducing BDNF synthesis and release of astrocytes through MEK/ERK pathway (23, 24). Furthermore, as a negative feedback mechanism, BDNF was found to be capable of reducing TNF-a levels (24). On astrocytes, two different TNF-α receptors (TNFR) activating MAP kinase as well as NF-ĸB pathways were found (23): TNFR1 mainly exerts inflammatory and pro-apoptotic processes, while TNFR2 exerts anti-inflammatory effects, proliferation of oligodendrocyte progenitor cells (OPC) and thus remyelination and neuronal survival. TNFR2 was also found to be capable of enhancing BDNF translation through activation of NF-ĸB and cyclic adenosine monophosphate (AMP)-response element binding protein (CREB) (23–25). Still, these mechanisms seem to be time dependent. Rowhani-Rad and colleagues found the early inflammatory processes in experimental autoimmune encephalomyelitis (EAE)-lesions to reduce BDNF through activation of TNFR1, with increasing TNF-a levels. Within 2 weeks after induction of the lesions, BDNF levels increased while TNF-a levels decreased through TFNR-2 activation (24).

The release of BDNF depends on the neuronal activity, though different neuropeptides and hormones can also influence the secretion. If glutamate is released from excitatory synapses, depolarization is caused by an influx of Ca^2+^ and Na^+^ via α-amino-3-hydroxy-5-methyl-4-isoxazolepropionic acid (AMPA), N-methyl-D-aspartate (NMDA) and voltage-dependent Ca^2+^-channels. This causes calcium-dependent activation of Ca^2+^-calmodulin-dependent protein kinases (CaMK), protein kinase C (PKC), and mitogen-activated protein kinases (MAPKs), which in turn leads to transcription of the BDNF gene by CREB and nuclear factor ĸB (NF-ĸB). After transcription, BDNF is packaged in vesicles and transported axonally to the dendrites and presynaptic endings, where it can be released through the activation of glutamate receptors (26). A detailed description of the synthesis and release of BDNF was reviewed by Kowiański et al. (27) as well as Marosi and Mattson (26).

### 1.2 BDNF signaling pathways

After release, BDNF binds to its two receptors: TrkB receptor and neurotrophin receptor p75 (nt-p75). The nt-p75 receptor, as a member of the tumor necrosis factor (TNF) family, provides pro-apoptotic functions. However, because of its low affinity to BDNF, expression of ceramides, NF-ĸB, and jun-kinases leading to apoptosis only will be activated at higher concentrations (28). It is hypothesized that because of increasing BDNF secretion in inflammatory lesions with a high density of these receptors at the edge of inflammatory lesions, cell death will be initiated in case of severe neuronal damage.

**Figure 1:**
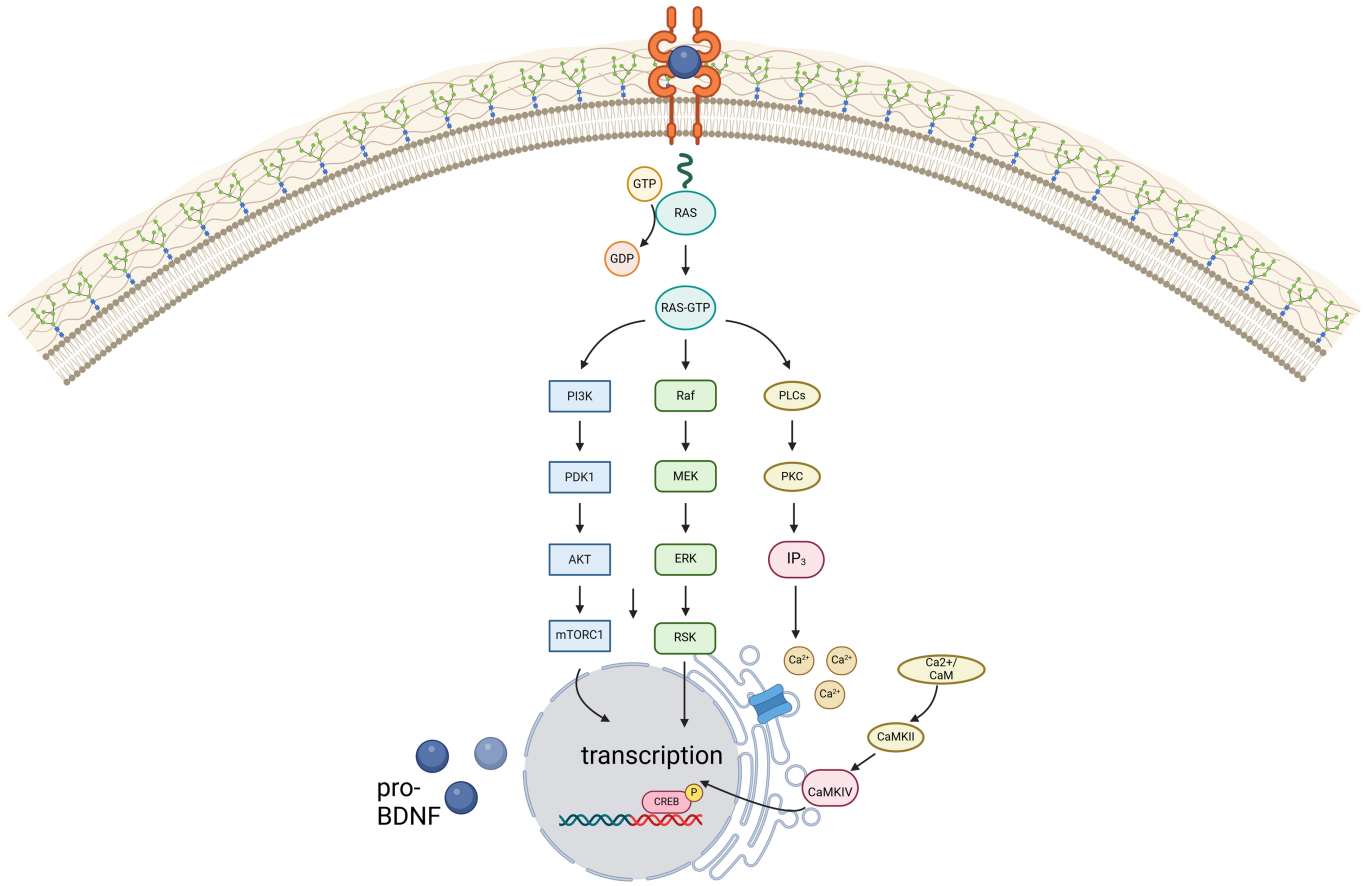
Illustration of the three main signaling pathways of BDNF, mediated via the high-affinity receptor TrkB. Created in BioRender.com.

Contrarily, due to its high affinity for BDNF, TrkB is activated even at low concentrations of BDNF, thereby activating phospholipase C gamma (PLC-γ), phosphatidylinositol-3 kinase (PI3-K) and MAP kinase pathway, illustrated in Figure 1. For remyelination, the proliferation, migration, and maturation of OPC to mature oligodendrocytes forming new myelin sheaths is crucial. Among others, intrinsic signaling cascades involving mTOR activation were shown to be relevant for this process (29). mTOR – a serine/threonine kinase - binds different proteins to form two protein complexes referred to as mTORC1 and mTORC2 (30). For synaptic plasticity, particularly mTORC1, consisting of mTOR as well as two proteins called raptor and GbL, is relevant (31, 32). By activation of the ribosomal protein S6 kinase (p70S6K), mTORC1 induces translation of proteins related to synaptic formation as well as excitatory postsynaptic currents (31), outgrowth of cytoskeletal filaments, axons as well as dendrites and dendritic spines (31, 33). Furthermore, the PI3K signaling pathway controls suppression of pro-apoptotic factors as Bad, Forkhead and thus reducing Fas ligand (33). Synergistically, activation of the MEK/ERK signaling pathway induces translation of the anti-apoptotic Bcl-2 (33). mTORC1 also inhibits autophagy through inhibition of ULK1, thus exerting further neuroprotective effects (31). However, as BDNF can activate these pathways through TrkB-receptors, BDNF is hypothesized to be a key factor promoting maturation of OPCs. Wong et al. could show, that in TrkB knock-out mice myelin protein expression, myelination as well as proliferative potential of OPCs was significantly decreased, thus proving that TrkB receptors are expressed on OPCs (34).

However, the increased expression of antioxidant enzymes and improved DNA repair mechanisms also contribute to the survival of neurons. But not only the survival of neurons is supported by BDNF: by activating p21-ras, BDNF can stimulate neurite outgrowth and synaptogenesis, increase cytoskeletal dynamics, and modulate cell adhesion (23). Furthermore, the NMDA-receptor subunits NR1 and early growth response factor-3 mediated mechanisms can be increased by BDNF via CREB, being hypothesized to improve cognition by increasing Ca^2+^ influx in synapses involved in learning and memory (23). Therefore, BDNF plays a pivotal role in long-term potentiation. Additionally, as BDNF can increase the synthesis of the peroxisome proliferator-activated receptor gamma coactivator 1-alpha (PGC-1α) as well as the transcriptional factor FOXO3a, with both playing a key role in the cellular energy homeostasis, maintenance, and formation of synapses is enhanced (23, 35).

### 1.3 BDNF in context of MS

Because of these effects, BDNF has gained increasing attention in the context of MS, especially regarding possible effects on clinical improvement by its remyelinating mechanisms (36, 37). Interestingly, decreased (38–42) as well as increased (39, 43–46) or normal (37, 47, 48) BDNF levels were found in patients with MS (pwMS) compared to healthy controls (49). Still, most studies reported an increase of BDNF concentrations in presence of disease activity as clinical relapse (37, 39, 40, 42, 46, 50). Decreasing BDNF concentrations with disease progression may be linked to decreased BDNF secretion of lymphocytes, as a change of lymphocyte phenotype was found with disease progression (14). Still, some studies found no association of low BDNF levels with disease progression (51, 52).

To date, knowledge of the role of BDNF in the field of MS is scarce as studies have been inconclusive so far. Accordingly, this review aimed to analyse the current literature regarding BDNF in different areas of multiple sclerosis and to provide an overview of the current state of knowledge in this field.

## 2 BDNF-polymorphism

BDNF is encoded on chromosome 11 band p13-14 (53). Only a few genetic variants were discovered in the coding intron regions, with the Val66Met polymorphism being the best-studied variant to date. In this single nucleotide polymorphism (SNP), the amino acid valine is exchanged for methionine in codon 66 (54). Despite this polymorphism, a functional protein with an altered pro-domain is synthesized, which, however, leads to changes in protein folding and thus reduced binding to TrĸB (55), but also to changes in the interaction with various other proteins (54). It has been shown that the binding behavior of translin, a DNA-binding protein, to BDNF messenger ribonucleic acid (mRNA) is disrupted, resulting in impaired trafficking of BDNF transcripts within dendrites (56). Additionally, it is hypothesized that the SNP results in altered intracellular packing and transport (1, 55, 57). Both *in vitro* studies and studies in healthy volunteers have shown that the altered intracellular transport leads to an abnormal secretion, thereby reducing the activity-dependent BDNF concentration by 18-30% (1, 55, 57). Some studies could not find a reduction of peripheral BDNF levels, which is why a local influence of activity-dependent secretion by the polymorphism has been postulated (45, 58). Altered secretion of a pro-apoptotically active precursor of BDNF due to altered intracellular trafficking (55, 57, 59) and alteration of the rate at which pro-BDNF is cleaved extracellularly by enzymes into mature BDNF have also been shown to be associated with the SNP (60). However, the reduced activity-dependent secretion has also been confirmed in Val66Met knock-in mice (61).

Nonetheless, studies remain inconclusive with some showing higher (62, 63) and some showing lower (57, 64) or equal (65, 66) BDNF-serum concentrations in healthy Val66Met carriers, whereas RRMS patients with Met alleles had higher BDNF concentrations than healthy carriers (45). However, some studies did not find differences in the BDNF serum concentration between healthy controls and RRMS patients with Val66Met polymorphism (45, 52, 67, 68). Studies also showed different results about peripheral mRNA levels, on the one hand with no detectable difference between Val homozygotes and Met carriers in either healthy subjects or RRMS patients (52, 67, 68), but on the other hand higher mRNA levels in RRMS patients compared to healthy Met carriers (45, 54).

## 3 BDNF and cognition

BDNF is thought to play a central role in synaptic plasticity (36, 69). It is hypothesized to play an important role in the long-term potentiation and neuroplasticity of the hippocampus (70), which is essential for learning and memory (71, 72). Several pathways how BDNF could affect episodic memory are discussed. However, increased activation of NMDA and AMPA-receptors and therefore increased intracellular calcium as well as sodium and potassium concentrations due to enhancement of protein translation via TrkB is hypothesized to play a key role (72, 73).

However, studies assessing the relationship between serum BDNF concentrations and cognitive performance have been inconclusive so far.

In animal models, low BDNF concentrations resulted in cognitive deficits due to disturbed neurotransmission and neuroplasticity (5, 74–76). In pwMS, a relationship between BDNF concentrations and cognitive performance was assumed (77). It was considered that the pathological changes leading to cognitive impairment were compensated through hippocampal hyperactivation to preserve episodic memory as seen in functional MRI (78, 79), which could be driven by BDNF (55, 80–83). Some studies reported a correlation of high BDNF levels with improvement of memory (84–86) and correlation of decreased BDNF concentrations with mild cognitive impairment even in newly diagnosed pwMS with no or mild disability (36). A study analysing changes in BDNF concentration after physical activity for 1 year found increasing BDNF levels with improvement of cognitive functions (87, 88). In reverse, a lack of BDNF or a disruption of the BDNF-TrkB pathway led to loss of synapses and decreasing cognitive functions (69, 89).

Regarding the effects of the Val66Met SNP on cognition, it is hypothesized, that the SNP prevents the development of maladaptive cortical plasticity in pwMS, resulting in improved recovery after relapses compared to Val homozygotes (90). A deleterious effect of the polymorphism on the parieto-prefrontal network and hippocampal activity, however, has been demonstrated in healthy subjects, whereas the opposite was found in RRMS patients (91). Val homozygote RRMS patients showed higher activity in the episodic memory network, maybe as form of compensatory mechanisms (92). Furthermore, pwMS with Val66Met polymorphism showing altered hippocampal activity while performing an episodic memory task (81, 82) had poorer episodic memory and executive functions (49, 81, 93). Still, some studies did not find any association of the Val66Met polymorphism with cognitive impairment in pwMS (45, 60, 94).

To summarize, some studies concluded that BDNF influences cognition, rather on a global level than influencing specific cognitive domains (86, 95), while other studies did not find an association between BDNF levels and cognition. Furthermore, most studies reported no detrimental effect of the Val66Met polymorphism on cognition. Table 1 provides an overview of the above-mentioned studies investigating the relationship between BDNF and cognition.

**Table 1:** Overview of the above-mentioned studies investigating the relationship between BDNF and cognition.

Study	Participants	Parameters	Results
Castrén et al. (84)	Animal study (rats)	Hippocampal mRNA expression of BDNF (among others) before and after induction of long-term potentiation (LTP) through stimulation of the perforant pathway.	Increase of BDNF mRNA in granular neurones of the dentate gyrus of both hemispheres by unilateral stimulation.
Cerasa et al. (91)	29 patients with RRMS, 32 healthy controls (HC)	Effect of Val66Met polymorphism on brain activity during spatial working memory task measured by fMRI	Increased activation of the parieto-prefrontal network and decreased activation of the ventro-medial-prefrontal cortex and hippocampus in healthy controls, no effect in RRMS-patients.
Ehling et al. (95)	19 patients with RRMS (12 treated with glatiramer acetate, 7 treatment-naïve)	Serum BDNF over 24 months	No difference between patients treated with glatiramer acetate and treatment-naïve patients and no change of BDNF serum levels over time
Engin et al. (87)	Animal study (mice)	Effect of α5-containing GABAa receptor knockout in dentate gyrus on cognition	Reduced tonic inhibition in knockout micee resulting in similar or better performance of memory tasks Impairment in cognitive tasks requiring formation of new/overwriting old circuits
Falkenberg et al. (85)	Animal study (rats)	Association of BDNF with motor activity, environment and cognition	Association of higher expression of BDNF mRNA in hippocampus with improved spatial memory and enriched environment
Fera et al. (92)	26 RRMS patients without cognitive impairment, 25 healthy controls	Effect of Val66Met polymorphism on hippocampal function in fMRI during episodic memory task	Higher activity parahippocampal, in left posterior hippocampus and left posterior cingulate cortex during encoding and retrieval as well as higher connectivity between hippocampus and posterior cingulate cortex during retrieval in Val homozygote RRMS patients opposite effects in healthy controls
Giordano et al. (90)	100 patients with progressive MS	Effects of BDNF and NTRK2 genes on motor recovery	In Val66Met carriers greater motor improvement after rehabilitation
Hakansson et al. (88)	23 healthy participants (19 fully participated)	35 min of aerobic exercice at moderate level, cognitive training through a computerized working memory training program or mindfulness practice using an app	Increase of serum BDNF levels after aerobic training, but not after cognitive training oder mindfulness practice Positive association of serum BDNF levels and working memory function
Hariri et al. (81)	64 healthy participants	Association of Val66met genotype and hippocampal activity in fMRI during declarative memory task	Decreased hippocampal activity in Val66Met carriers compared to Val66Val during encoding and retrieval processes
Hulst et al. (78)	34 patients with MS without cognitive impairment, 16 patients with MS with cognitive impairment, 30 healthy controls	fMRI during episodic memory task	Bilaterally increased brain activity parahippocampal and left anterior cingulate gyrus in patients without cognitive impairment during encoding, less brain activity para−/hippocampal and in the prefrontal cortex in cognitively impaired patients, but increased activity in posterior cingulate gyrus and left precuneus No structural differences between patients with and without cognitive impairment
Islam et al. (70)	Animal study (mice)	Effect of BDNF on multipotent neural stem cells	Modulation of neurogenesis by mainly mediated via TrkB, a.o. by regulating neural replacement, neural loss while
Loprinzi (73)	Metaanalysis	52 studies—26 animal studies, 15 studies in humans	All animal studies showed improvement of memory, 97% increase of BDNF with 8 studies showing BDNF as mediator of the positive association of training and cognition. 44% of the studies in humans found an positive association of BDNF and cognitive improvement with 40% of these assuming BDNF as mediator
Liguori et al. (45)	36 inactive patients with RRMS and 37 healthy controls	Effect of Val66Met polymorphism and BDNF levels on patients with RRMS over 24 months	Higher BDNF levels in patients with RRMS compared to healthy controls, regardless immunotherapy No correlation of Val66Met polymorphism with clinical or MRI characteristics
Linnarsson et al. (75)	Animal study (mice)	Effect of deletion of one copy of the BDNF gene	Significant cognitive impairment
Ninan et al. (83)	Animal study (mice)	Properties of hippocampal CA3-CA1 synapses of BDNF Met/Met mice and matched wild-type mice	Normal glutamatergic transmission, but decreased NMDA receptor-dependent transmission and long-term potentiation in BDNF met/met mice
Patanella et al. (77)	30 patients with RRMS	PBMC production of BDNF, TNF-alpha, IL10 and IL6	Association of low BDNF levels with worse performance in divided attention and visual scanning tasks Association of high IL-6 level with low mini mental state examination scores
Prokopova et al. (36)	19 treatment naïve patients with MS, 19 healthy controls	BDNF plasma level, cognitive function as well as effect of mild stress (Stroop test) on neuroendocrine activation in early phase of multiple sclerosis	Association of cognitive impairment in male patients with MS with decreased BDNF plasma levels Higher anxiety, depression and poorer performance in Stroop test in patients with MS
Ramasamy et al. (60)	188 patients with MS	Effect of Val66Met polymorphism on regional grey matter in MRI	Higher grey matter volume in the cingulate of patient with MS with Val66Met polymorphism compared to Val66Val patients
Vigers et al. (76)	Animal study (mice)	Quantification of dendritic spines in cortical neurons in late-onset forebrain-specific BDNF knockout mice	Smaller brain volume in knockout mice compared to wild-type mice with impairment in spatial learning and increased depression
Yalachkov et al. (86)	36 patients with RRMS, PPMS, CIS	Effect of BDNF on disability and cognition after relapse	Association of disability improvement with higher serum BDNF levels and cognitive improvement with higher BDNF levels in CSF at baseline with a positive correlation between serum BDNF and EDSS improvement and CSF-BDNF with *z*-score change in cognitive tests
Zivadinov et al. (1)	209 patients with MS (108 with cognitive testing)	Effect of Val66Met polymorphism on brain morphometry and functionality measured by MRI and cognitive testing	Positive association of Val66Met polymorphism with normalized grey matter volume and negative association with T2 lesion volume only trend towards a positive association

## 4 BDNF and disability

Studies assessing the effect of BDNF on disease severity, disability improvement, or worsening are scarce.

Some studies found no relationship between the BDNF concentration and the disease severity assessed by multiple sclerosis Severity Score (96). Contrarily, in a pilot study, Yalachkov and colleagues found that higher serum BDNF concentrations at baseline were associated with a disability improvement measured as an EDSS decrease of ≥0,5 12 months after relapse (86).

However, most studies focused on the relationship between the disease severity and the BDNF Val66Met polymorphism. No differences between Val66Met carriers and Val homozygotes were found regarding disease susceptibility (58, 94, 97), age at onset or disease severity (1, 49, 97, 98) assessed by EDSS (45, 94, 97, 98). Still, Val66Met carriers showed greater motor recovery after rehabilitation assessed by 6 min walk test (6-MWT) (90). Lower methylation of the BDNF gene was associated with higher gene expression and a higher risk of achieving an EDSS of 6,0 (99, 100). Only one study found susceptibility and disease outcome to be influenced by the polymorphism, especially in females (101). Furthermore, no association of the Val66Met polymorphism was found for disease duration, relapse rate, time between relapses, or time to reach EDSS milestones like 4 or 6 or secondary progression. To this end, no detrimental effect of the SNP was found in pwMS (98, 99).

However, further studies with larger sample sizes and long-term follow-up are needed to assess the effect of BDNF on disease activity and severity, as studies are scarce and results inconclusive so far. A summary of the studies assessing the relationship between BDNF and disability can be found in Table 2.

**Table 2:** Overview of the above-mentioned studies investigating the relationship between BDNF and disability.

Study	Participants	Parameters	Results
Blanco et al. (98)	224 patients with MS patients, 177 healthy controls	Effect of Val66Met polymorphism on disease susceptibility and severity	No association of Val66Met polymorphism with disease susceptibility or severity No effect of Val66Met polymorphism on peripheral levels BDNF in a subgroup of 12 patients with MS
Dinacci et al. (58)	45 patients with MS, 34 healthy controls	Association of Val66Met polymorphism with MRI characteristics	Lower grey matter volume in Val66Val patients compared to healthy controls, but not in Val66Met patients compared to healthy controls
Giordano et al. (90)	100 patients with progressive MS	Effects of BDNF and NTRK2 genes on motor recovery	In Val66Met carriers greater motor improvement after rehabilitation
Islas-Hernandez et al. (96)	74 patients with RRMS, 11 patients with SPMS, 88 healthy controls	Association of total Tau and BDNF with disease severity/disability	Increased tau and decreased BDNF in patients with MS Higher total tau in patients with RRMS and higher MSSS and lower EDSS scores, no differences in SPMS and patients with severe MS compared to healthy controls
Liguori et al. (49)	50 patients with RRMS, 50 healthy controls	Effect of Val66Met on MRI characteristics and cognition	Lower grey matter volume in patients withVal66Met polymorphism No association of Val66Met polymorphism with cognition in patients with RRMS and healthy controls
Liguori et al. (45)	36 inactive patients with RRMS and 37 healthy controls	Effect of Val66Met polymorphism and BDNF levels on patients with RRMS over 24 months	Higher BDNF levels in patients with RRMS compared to healthy controls, regardless immunotherapy No correlation of Val66Met polymorphism with clinical or MRI characteristics
Lindquist et al. (97)	951 British MS patients	Association of Val66Met polymorphism with disease susceptibility and disease severity	No association of Val66Met polymorphism with disease susceptibility or clinical course
Mero et al. (94)	2,149 patients with MS, 2747 helathy controls	Association of Val66Met polymorphism with disease susceptibility, demographical and clinical parameters	No association of Val66Met polymorphism with disease susceptibility, sex, age at onset, disease course and severity, cognitive impairment
Mirowska-Guzel et al. (101)	230 patients with RRMS/SPMS and 177 healthy controls	Association of BDNF gene (C270T and G196A) and disease susceptibility and disease progression	Earlier disease onset in male patients with 196G/G than 196G/A genotype Higher disease susceptibility in female patients with as well as 196G/G genotype in male patients with 270C/T genotype
Nociti et al. (99)	209 patients with MS	Association of Val66Met polymorphism with methylation level of the BDNF gene and disability	Association of higher disability/disease progression and hypomethylation of BDNF gene with higher BDNF levels
Portaccio et al. (100)	98 patients with MS	Association of Val66Met polymorphism with disability and cognition	Higher prevalence of Val66Met polymorphism in male patients and patients with greater disability Positive association of Val66Met polymorphism with better cognitive performance and higher EDSS
Yalachkov et al. (86)	36 patients with RRMS, PPMS, CIS	Effect of BDNF on disability and cognition after relapse	Association of disability improvement with higher serum BDNF levels and cognitive improvement with higher BDNF levels in CSF at baseline with a positive correlation between serum BDNF and EDSS improvement and CSF-BDNF with *z*-score change in cognitive tests
Zivadinov et al. (1)	209 patients with MS (108 with cognitive testing)	Effect of Val66Met polymorphism on brain morphometry and functionality measured by MRI and cognitive testing	Positive association of Val66Met polymorphism with normalized grey matter volume and negative association with T2 lesion volume onyl trend towards a positive

## 5 Effect of BDNF on MRI parameters

Again, more studies analysed the association of the Val66Met polymorphism to MRI characteristics in pwMS, compared to only few studies assessing the association of these to BDNF levels.

Comini-Frota and colleagues examined the correlation between BDNF and T2-lesion number in 28 patients with RRMS compared to 28 healthy controls. While they found BDNF levels correlating negatively with the number of lesions, BDNF levels did not correlate with T1 hypointense lesions or the presence of gadolinium-enhancing lesions. The authors concluded that as BDNF controls remyelinating processes, no correlation with T1 lesions as chronic lesions was found (102). However, this argumentation does not fully explain the missing correlation between BDNF and gadolinium-enhancing lesions as they display acute inflammatory lesions (102). Furthermore, another study found a negative correlation of serum BDNF levels with T1 lesion volume as well as quotient of T1 lesion volume to total lesion volume, displaying the relative contribution of T1 lesion volume to the total lesion volume, while no correlations were found for CSF BDNF level (103). In contrast, Weinstock-Guttman and colleagues found an association between higher BDNF concentrations and higher inflammation in white matter (WM), correlating with a higher WM volume. Moreover, they found that higher BDNF is associated with microscopic damage in the normal-appearing WM (NAWM) and concluded that BDNF may play a role in the early formation of inflammatory lesions or that diffuse inflammatory infiltrates without correlate on MRI are the pathological explanation for this (52). Regarding magnetization transfer ratio (MTR), the study found a negative correlation with increased BDNF, while no correlation between grey matter (GM) volume and normal appearing GM (NAGM) MTR was found with BDNF-levels (52), which might indicate that BDNF secretion only increases in inflammatory tissue (37).

However, more studies investigated the relationship between Val66Met polymorphism with MRI changes.

Though some studies reported a higher risk of GM atrophy in pwMS with Val66Met polymorphism (49, 91), most studies reported a protective effect of the SNP on brain atrophy (52, 58, 90) as well as GM atrophy (1, 52, 58, 60). Though higher GM volume was found throughout the brain in pwMS with Val66Met polymorphism, higher volume was especially found in the cingulate (1, 60), inferior frontal gyrus, parahippocampal gyrus, middle temporal gyrus, precentral gyrus, and inferior parietal lobule of the left cerebral hemisphere, in the right cerebral hemisphere, in the precuneus and middle frontal gyrus (1), consistent with regions of pronounced expression of BDNF (104, 105). As the highest atrophy related to the lesion volume in pwMS was found in the anterior cingulate (106) especially on the left hemisphere (107), a local influence of the polymorphism on BDNF secretion and thus on local inflammation has also been discussed to have a protective effect on regional brain volume in pwMS (58). Decreased cortical thickness was found in one study (108), thus a detrimental effect of the SNP was hypothesized. In another study, higher hippocampal volumes were found in pwMS with Val66Met polymorphism, compared to Val homozygotes (109). Therefore, protection of hippocampal tissue was assumed (109). Moreover, an association of the Val66Met polymorphism was found with lower T2-lesion volume (1) in comparison with Val66Val patients, though other studies did not find differences between groups in T2 or T1 lesion volume (45, 52, 108). Matching these structural imaging findings, increased functional connectivity between the hippocampus and posterior cingulate cortex was found in pwMS with Val66Met polymorphism during an episodic memory task (92). Still, two other studies reported a negative impact of the SNP polymorphism on functional activity of the brain. While Egan and colleagues showed an altered hippocampal activation (55), another study showed altered functional connectivity extending the hippocampal system with decreased functional connectivity in the ventromedial PFC and ACC-networks (91).

Overall, the study results indicate lower rates of brain atrophy and less pronounced signs of damage associated with BDNF. Regarding the Val66Met SNP, most studies brought evidence that the SNP has different effects on MRI measures in healthy controls compared to pwMS with the majority of studies assuming a protective effect of Val66Met polymorphism in pwMS. An overview of the above-mentioned studies can be found in Table 3.

**Table 3:** Overview of the above-mentioned studies investigating the relationship between BDNF and MRI characteristics.

Study	Participants	Parameters	Results
Brod et al. (103)	13 patients with active MS without immunotherapy	Association of serum and CSF biomarkers with MRI characteristics	Association of increased CSF neuronal cell adhesion molecules (NCAM) with T1 activity and of CSF and serum NCAM with T1 lesion volume Negative association of serum BDNF and relative contribution of T1 lesion volume to the total lesion volume
Cerasa et al. (91)	29 patients with RRMS 32 healthy controls (HC)	Effect of Val66Met polymorphism on brain activity during spatial working memory task measured by fMRI	Increased activation of the parieto-prefrontal network, disengagement of the ventro-medial-prefrontal cortex and hippocampus in HCs, no effect in RRMS patients
Charil et al. (106)	425 patients with early RRMS	Association of cortical thickness with other clinicoradiological characteristics	Association of cortical thickness with lesion load and disability, especially in cingulate gyrus, insula and associative cortical regions
Comini-Frota et al. (102)	28 patients with MS, 28 healthy controls	Association of BDNF and MRI characteristics	Decreased BDNF levels in patients with MS with a negative association to T2/FLAIR lesion load
Conner et al. (104)	Animal study (adult rats)	Detailed mapping of BDNF immunoreactivity and synthesis	Synthesis of BDNF in neurons with anterograde axonal transportand storage in axonal terminals
Dinacci et al. (58)	45 patients with MS, 34 healthy controls	Association of Val66Met polymorphism with MRI characteristics	Lower grey matter volume in Val66Val patients compared to healthy controls, but not in Val66Met patients compared to healthy controls
Dolcetti et al. (108)	218 patients with RRMS	Association of Val66Met polymorphism and CSF levels of pro- and anti-inflammatory molecules with clinicoradiological characteristics	Association of Val66Met polymorphism and the proinflammatory molecules MCP-1, IL-8, TNF, Eotaxin, and MIP-1b and cortical atrophy at time of diagnosis, but not with clinical characteristics
Fera et al. (92)	26 RRMS patients without cognitive impairment, 25 healthy controls	Effect of Val66Met polymorphism on hippocampal function in fMRI during episodic memory task	Higher activity parahippocampal, in left posterior hippocampus and left posterior cingulate cortex during encoding and retrieval as well as higher connectivity between hippocampus and posterior cingulate cortex during retrieval in Val homozygote RRMS patients opposite effects in healthy controls
Giordano et al. (90)	100 patients with progressive MS	Effects of BDNF and NTRK2 genes on motor recovery	Greater motor improvement after rehabilitation in Val66Met carriers
Liguori et al. (49)	50 patients with RRMS, 50 healthy controls	Effect of Val66Met on MRI characteristics and cognition	Lower grey matter volume in patients withVal66Met polymorphism No association of Val66Met polymorphism with cognition in patients with RRMS and healthy controls
Liguori et al. (45)	36 inactive patients with RRMS and 37 healthy controls	Effect of Val66Met polymorphism and BDNF levels on patients with RRMS over 24 months	Higher BDNF levels in patients with RRMS compared to healthy controls, regardless immunotherapy No correlation of Val66Met polymorphism with clinical or MRI characteristics
Meo et al. (109)	50 patients with MS, 15 healthy controls	Effect of Val66Met polymorphism on hippocampal subfield volumes and cognition	Patients with MS had lower volume of hippocampus-amygdala transition area, cornus ammonis (CA1), granule cell layer of dentate gyrus, CA4 and CA3 of left hippocampal head, molecular layer of the left hippocampal body; presubiculum of right hippocampal body and right fimbria Val66Met polymorphism was associated with higher volume of hippocampal tail; CA1, ML, CA3, CA4, and GCL-DG of left hippocampal head; CA1, ML, and CA3 of the left hippocampal body; left hippocampus-amygdala transition area, presubiculum of the right hippocampal headNegative association of higher lesion load with lower volume of presubiculum of right hippocampal body Positive association of left hippocampal tail volume withvisuospatial memory and left hippocampal head volume with semantic fluency
Prinster et al. (107)	188 patients with MS	MRI characteristics and association with disease duration and severity	Decreased grey matter volume of left fronto-temporal cortex and precuneus, anterior cingulate gyrus, bilateral caudate nuclei, cortical grey matter of postcentral area in patients with MS
Ramasamy et al. (60)	188 patients with MS	Effect of Val66Met polymorphism on regional grey matter in MRI	Higher grey matter volume in the cingulate of patient with MS with Val66Met polymorphism compared to Val66Val patients
Sarchielli et al. (37)	20 patients with RRMS (7 with acute relapse), 15 patients with SPMS, 20 healthy controls	Association of BDNF production by PBMCs unstimulated and stimulated with phytohemagglutinin, anti-OKT3 AB and MPB with clinicoradiological characteristics	Patients with MS had lower volume of hippocampus-amygdala transition area, cornus ammonis (CA1), granule cell layer of dentate gyrus, CA4 and CA3 of left hippocampal head, molecular layer of the left hippocampal body; presubiculum of right hippocampal body and right fimbria Val66Met polymorphism was associated with higher volume of hippocampal tail; CA1, ML, CA3, CA4, and GCL-DG of left hippocampal head; CA1, ML, and CA3 of the left hippocampal body; left hippocampus-amygdala transition area, presubiculum of the right hippocampal head Higher BDNF in supernatants of unstimulated and stimulated PBMCs in RRMS patients during and after relapse compared to stable phaseLower BDNF in unstimulated and stimulated PBMC supernatants of patients with SPMS compared to healthy controls, especially in patients with recent EDSS worsening (≥1 point in the last 6 months) Positive association of left hippocampal tail volume withvisuospatial memory and left hippocampal head volume with semantic fluency
Weinstock-Guttman et al. (52)	52 patients with relapsing MS	Association of BDNF with clinical and MRI variables	Positive association of BDNF with contrast-enhancing lesion volume and higher white matter volume as well asnegative Association of MTR of contrast-enhancing lesion volume and normal-appearing white matter with BDNF, both after stimulation with anti-CD3 and anti-CD28; Decreasing BDNF secretion with increasing disease duration; No association with Val66Met polymorphism
Zivadinov et al. (1)	209 patients with MS (108 with cognitive testing)	Effect of Val66Met polymorphism on brain morphometry and functionality measured by MRI and cognitive testing	Positive association of Val66Met polymorphism with normalized grey matter volume and negative association with T2 lesion volume only trend towards a association of Val66Met polymorphism with PASAT

## 6 Effect of immunotherapies on BDNF

Immunotherapies have been hypothesized to modulate the disease course not only by exerting anti-inflammatory effects but also neuroprotective and neuroregenerative mechanisms. Therefore, the correlation of immunotherapies with BDNF was investigated in several studies. So far, especially interferon-beta (IFN- β) and glatiramer acetate have been analysed.

For glatiramer acetate, higher BDNF levels were found in pwMS (39, 45, 46, 110–112). In an *ex-vivo* study, a significant increase of BDNF levels in supernatants of unstimulated peripheral blood mononuclear cells (PBMC) was found 6 months after initiation of glatiramer acetate treatment, supported by studies analysing glatiramer acetate specific T-cell lines (110). As a possible mechanism, a shift of pro-inflammatory T helper (T_H_) 1 cells to anti-inflammatory T_H2_ cells, which is the main therapeutic action of glatiramer acetate, is thought to promote neuroregenerative mechanisms due to BDNF induction and release (110, 113). Furthermore, besides anti-inflammatory T_H2_-cytokines, glatiramer acetate-specific cells were thought to express BDNF themselves (95, 114, 115). This is further supported by animal studies also providing evidence that glatiramer acetate exhibits neuroprotective effects through BDNF. In EAE, increased BDNF levels were found after treatment initiation (39, 111, 112, 116–118) as well as an augmentation of BDNF expression in histological glatiramer acetate treated EAE (116) with reduced axonal damage and decreased neurodegeneration (119). Moreover, in EAE the clinical efficacy of glatiramer acetate was limited after BDNF deletion (120). However, other studies showed no increase of BDNF levels in pwMS treated with glatiramer acetate (50, 95, 121).

In clinically stable patients with INF-β treatment, significantly increased BDNF levels were found (38, 46) compared to treatment naïve patients, sustained after 6 months as well as a trend to increased level after 1 year in RRMS, but not in SPMS-patients (43). Though patients treated with INF-ß showed higher BDNF levels in relapse-free intervals compared to healthy controls, no additional increase of BDNF was found in relapses (42). Moreover, patients treated with IFN-β not only had higher BDNF levels compared to treatment naïve patients but also in comparison to patients treated with mitoxantrone with an inverse correlation between BDNF levels and degree of disability (51). Contrary to this, some studies reported no effect of IFN-β on BDNF levels (15, 43, 45, 122). In a mixed cohort with 38 subjects treated with IFN-β and 12 with glatiramer acetate, no significant increase of BDNF levels was found after 1 year, with no differences between treatment groups (123).

Studies investigating the effect of other immunotherapies than INF-ß and glatiramer acetate are rare. However, Golan and colleagues showed a significant increase of BDNF in patients treated with fingolimod after 6 and 12 months (121), which is in line with a previous animal study, showing that the neuroprotective effect of Fingolimod is associated with the induction of the BDNF production (124). One study also could show increased BDNF levels in RRMS-patients treated with natalizumab (125). Moreover, significantly lower BDNF concentrations in patients treated with high-dose immunoglobulins were found, possibly due to interference of immunoglobulins with neuroprotective mechanisms (122). Regarding the effects of therapy of acute relapses with glucocorticoids, increased BDNF levels could be shown in EAE (95), but could not be confirmed in humans (37, 42).

To summarize, most studies hypothesized increased BDNF concentrations in patients treated with glatiramer acetate and IFN-β, while the number of studies in patients with other immunotherapies was too small to draw a conclusion. For details of the respective studies, see Table 4.

**Table 4:** Overview of the above-mentioned studies investigating the relationship between BDNF and immunotherapies.

Study	Participants	Parameters	Results
Aharoni et al. (117)	Animal study (mice)	Expression of TH_2/3_ cytokines and neurotrophic factors in glatiramer acetate specific T-cells	Increased levels of BDNF and IL-10 and transforming growth factor by astrocytes and microglia (among others) in glatiramer acetate treated mice
Aharoni et al. (116)	Animal study (mice)	Production of BDNF and other neurotrophins, as well as histopathology under glatiramer acetate treatment	Increased neurotrophins in treatment-naïve mice after EAE induction with lower concentrations over time compared to control mice Increased levels of BDNF, NT2 and NT3 in various brain regions under glatiramer acetate treatment with particularly high lesional concentration by migration of neuronal progenitors
Azoulay et al. (39)	74 patients with RRMS (26 patients treated with IFN, 27 with glatiramer acetate, 21 without immunotherapy), 28 healthy controls	Measurement of BDNF (ELISA)	Lower BDNF levels in blood and CSF in patients with RRMS, increased in patients treated with glatiramer acetate Lower BDNF levels in patients with RRMS with acute relapse compared to stable patients with RRMS Only trend to higher BDNF levels in patients treated with IFN-β
Azoulay et al. (38)	22 treatment-naïve patients with RR-MS, 27 IFN-β1a-treated RR-MS patients	BDNF in the supernatants of PBMCs (ELISA)	Higher BDNF levels in patients with RRMS treated with IFN-β1a, with upregulation by stimulation with anti-CD40
Blanco et al. (110)	19 treamtent-naïve patients with MS started on glatiramer acetate	Measurement of BDNF in unstimulated and stimulated (anti-CD3, anti-CD28) supernatants from PBMC by ELISA and intracellular cytokine production by flow Cytometry over 21 months	Decrease of BDNF production as well as INF-y producing total lymphocytes, CD4+ and CD8+ T cells, reduced percentage of IL2 producing CD4+ and CD8+ T-cells; increase of CD3+ and CD4+ and CD4 + CD45RA + T-cells after 6 months
Caggiula et al. (43)	25 patients with RRMS, 14 patients with SPMS treated with IFN-β	Production of BDNF, NGF, GDNF, NT3, NT4 by PBMCs	Increasing BDNF levels in clinical stable patients with RRMS after 6 months of IFN-β treatment
Chen et al. (111)	12 patients with MS treated with glatiramer acetate	BDNF production in different T-cell lines	Significant higher BDNF levels by resting T-cell lines Higher BDNF levels especially in glatiramer acetate stimulated T-cell lines
Ehling et al. (95)	19 patients with RRMS (12 treated with glatiramer acetate, 7 treatment-naïve)	Serum BDNF over 24 months	No difference between patients treated with glatiramer acetate and treatment-naïve patients and no change of BDNF serum levels over time
Golan et al. (121)	21 patients with MS	BDNF, GDNF, ß-NGF, NT-3, NT-4, FGF, EGF, VEGF (ELISA) before fingolimod treatment and after 6 and 12 months	Increaed BDNF levels after initiation of treatment with fingolimod
Kalinowska-Lyszczarz et al. (123)	50 treatment naïve patients with RRMS, 39 healthy controls	BDNF levels in PBMCs lysates over one year of immunomodulation with IFN-β or glatiramer acetate (ELISA)	Lower BDNF levels in patients with MS compared to healthy controls No change of BDNF levels under immunotherapy without difference between treatment groups
Lee et al. (120),	Animal study (mice)	Effect of glatiramer acetate in BDNF knock-out mice	Limited clinical efficacy of glatiramer acetate after BDNF deletion in early disease phases
Liguori et al. (45)	36 inactive patients with RRMS and 37 healthy controls	Effect of Val66Met polymorphism and BDNF levels on patients with RRMS over 24 months	Higher BDNF levels in patients with RRMS compared to healthy controls, regardless immunotherapy
Lindquist et al. (42)	15 patients with RRMS, 1 patient with SPMS during acute relapse	Cytokine levels (TNF-a, iNOS, CNTF, BDNF) in PBMCs (flow-cytometric approach)	No effect of glucocorticoids on BDNF levels Increased BDNF levels in patients treated with IFN-β
Mehrpour et al. (51)	82 patients with MS (15 treated with avonex, 13 with Rebif, 27 with Betaferon, 3 with Mitoxantrone, 2 with immunoglobulins, 22 without immunotherapies)	Serum BDNF levels (ELISA)	Higher BDNF levels in patients treated with IFN-β1b compared to patients treated with Mitoxantrone/patients without immunotherapies Negative correlation of BDNF with clinical impairment Patients with BDNF levels over 190 pg/ml indicated EDSS < 3 with 80% sensitivity
Petereit et al. (122)	27 patients with RRMS, 10 healthy controls	BDNF blood level	Higher BDNF level patients with MS compared to healthy controls IFN-β and low dose immunoglobulin had no effect on BDNF levels, while high-dose immunoglobulin decreased BDNF levels
Sarchielli et al. (37)	20 patients with RRMS (7 with acute relapse), 15 patients with SPMS, 20 healthy controls	Association of BDNF production by PBMCs unstimulated and stimulated with phytohemagglutinin, anti-OKT3 AB and MPB with clinicoradiological characteristics	Higher BDNF in supernatants of unstimulated and stimulated PBMCs in RRMS patients during and after relapse compared to stable phase Lower BDNF in unstimulated and stimulated PBMC supernatants of patients with SPMS compared to healthy controls, especially in patients with recent EDSS worsening (≥1 point in the last 6 months)
Sarchielli et al. (46)	60 patients with RRMS (20 patients treated with IFN-β, 20 patients with glatiramer acetate, 20 patients with high dose immunoglobulins)	Cytokine and BDNF production by PBMCs over 2 years stimulated with Phytohemagglutinin, anti-OKT3 antibody, myelin basic protein	Increasing BDNF levels over 3 months in patients with MS treated with glatiramer acetate, afterwards levels remained stable but significantly higher compared to controls Decreased IFN-g, IL4, IL5, IL10 in patients treated with glatiramer acetate No effect of IFN-β1a/minimal decrease of BDNF levels under immunoglobulin treatment Increase of IL10 in patients treated with IFN-β1a
Shajarian et al. (15)	45 patients with RRMS (32 treated with IFN-β, 13 treatment naïve), 45 healthy controls	Plasma BDNF and IL6 (ELISA)	Significant lower BDNF and higher IL6 in patients with MS compared to healthy controls No significant effect of IFN-β on BDNF plasma levels, but significant positive correlation of BDNF and IL-6
Smith et al. (124)	Animal study (mice)	Association of brain volume in 7 T MRI with BDNF levels in blood, cerebrospinal fluid	Progressive brain volume loss despite clinical stability Less atrophy of cerebellum and striatum in mice treated early with fingolimod due to increased BDNF levels No influence of teriflunomide on atrophy rates
Vacaras et al. (50)	19 patients with RRMS treated with glatiramer acetate, 11 patients with RRMS without immunotherapy, 12 healthy controls	BDNF in plasma, TrkB activation over 1 year	Reduced BDNF plasma level in patients with MS, no influence of glatiramer acetate after one year of sustained therapy
Vacaras et al. (125)	22 patients with RRMS (11 treatment naïve, 11 treated with natalizumab), 9 patients with SPMS, 20 healthy controls	BDNF in plasma (ELISA)	Higher BDNF levels in patients with MS treated with natalizumab
Ziemssen et al. (118)	Patient with MS treated with glatiramer acetate, Healthy control	Glatiramer acetate specific T-cell lines, BDNF reverse-transcription PCR and two different BDNF measurement methods	BDNF production of all glatiramer acetate specific T_H1_, T_H2_ and T_H0_ after stimulation
Ziemssen et al. (112)	Patients with MS treated with glatiramer acetate, healthy controls	Glatiramer acetate specific T-cell lines, BDNF reverse-transcription PCR and two different BDNF measurement methods	BDNF production of all glatiramer acetate specific TH1, Th2 and Tho cell lines after stimulation

## 7 Effect of autologous hematopoietic stem cell transplantation on BDNF

Autologous hematopoietic stem cell transplantation (HSCT) has received increasing attention in the field of MS therapy in the past few years. Although studies could show a sustained suppression of inflammation and therefore disease activity, the exact mechanisms of action remain unclear (126). Muraro and colleagues showed a significant increase of naïve CD4-positive T-cells two years after HSCT, suggesting the suppression of inflammatory activity in pwMS after HSCT does not depend on persisting suppression of lymphocytes, but qualitative immunological changes (126). Consequently, an increasing number of studies with follow-up data from large registries have shown positive effects on disease activity with stabilization (127), disease severity as measured by EDSS either with stabilization or even improvement (127), fatigue and quality of life (128). Positive effects of HSCT have also been demonstrated on paraclinical markers of disease activity such as new or enlarging T2 lesions or contrast-enhancing lesions (127). However, mostly patients with active RRMS or SPMS benefit from HSCT (129). In a study comparing immunotherapies and HSCT in 110 patients with RRMS, non-myeloablative HSCT resulted in prolonged time to disease progression (130).

Our literature research revealed only one study examining the effect of HSCT on BDNF levels in serum and CSF (127). In 14 pwMS, serum BDNF levels at baseline measurement were significantly higher compared to controls with a significant decrease over 12 months, almost at the level of healthy controls. Furthermore, a significant decrease of BDNF levels was also found in CSF, though CSF BDNF levels were only accessible in 9 patients. Possibly, the decreasing BDNF levels were due to the sustained suppression of CD4+ T-cells. However, only a positive correlation was found between CSF BDNF level and the T2 lesion load at baseline and at 12 month follow-up. Though one could assume a possible detrimental effect of decreased BDNF, no neurological deterioration nor correlations between BDNF and atrophy measurement were found. Blanco and colleagues provided evidence that BDNF levels decreased due to HSCT, possibly due to a shift of pro-inflammatory T_H1_ to anti-inflammatory T_H2_-cells, supporting the hypothesis that HSCT does not only has immunosuppressive but also immunomodulatory effects (127).

As HSCT becomes more and more relevant in the treatment of MS, it is likely that the number of studies analysing the immunomodulatory effects and its relationship with BDNF will increase over the next few years. Table 5 summarises the studies mentioned above.

**Table 5:** Overview of the above-mentioned studies investigating the relationship between BDNF and HSCT.

Study	Participants/Inclusion criteria	Results
Muraro et al. (126)	7 patients with MS with progression of 1.5/1 point/s in EDSS (when EDSS ≤6/≥6) in the last year before HSCT (2 years follow up)	Significant increase of naïve CD4+ cells with recent thymic origin and decrease of memory T-cells with a broader spectrum of clonal diversity and renewal of clone-specific characteristics after HSCT
Blanco et al. (127)	14 patients with MS treated with HSCT (3 years follow up)	Significant decrease of serum BDNF levels over 12 months after HSCT correlation of CSF-BDNF levels with T2 lesion load before and 12 months after HSCT
Giedraitiene et al. (128)	18 patients with highly active MS treated with AHSCT (2 years follow up)	Improvement of physical functioning, vitality, fatigue, pain, decrease of EDSS with positive impact on health related Quality of life
Mancardi et al. (129)	74 patients with MS treated with AHSCT (median of 48.3 months follow up)	66% patients remained stable or improved after 5 years 2 patients died
Burt et al. (130)	55 patients with MS treated with HSCT and 55 patients with MS treated with immunotherapies (5 years follow up)	Disease progression in 3 patients treated with HSCT and 34 patients treated with immunotherapies EDSS improvement in patients treated with HSCT, increase of EDSS in patients treated with immunotherapies

## 8 Influence of physical activity and exercise training on BDNF

In recent years, there has been increasing evidence that physical activity as well as exercise has positive effects on pwMS, exerting neuroprotective effects and thus slowing neurodegeneration and disease progression (131). It is hypothesized that BDNF – as well as other neurotrophins – are secreted in dependency of neuronal activity (132–134) and due to certain factors transmitted in the bloodstream from peripheral organs as skeletal muscles (135–137). Hence, muscle activity leads to increasing neurotrophin levels in the blood, ultimately migrating in the central and peripheral nervous system (135, 137). As a result, there have been numerous studies linking neuroprotective effects of exercise to neurotrophic factors such as BDNF (138, 139). In case of running, increasing intracellular Ca^2+^ concentrations and induction of CaMK via CREB were found to induce BDNF synthesis (23, 140). Still, it is controversial whether peripheral BDNF can pass the blood–brain barrier to induce effects in the CNS. Hakansson and colleagues found an immediate increase of serum BDNF levels after 35 min of physical activity, while participants doing cognitive training or mindfulness practice for 35 min showed no increases (88). A study assessing the effect of resistance training on BDNF within two hours after the training found no within and between-group differences before and no within-group differences after the 24 weeks long training intervention (141). Moreover, four other studies failed to assess within or between-group differences in BDNF serum level (142–145), while two studies only assessed within group differences with an increase of BDNF level during the exercise intervention (146, 147). However, the majority of studies assessed between-group differences favouring the exercise intervention (148–153). In a meta-analysis assessing the effects of exercise training on neurotrophic factors, the results for different neurotrophins (NGF, CNTF, IGF-1, NT3-5, GDNF, PDGF as well as VEGF) remained inconclusive, but for BDNF a significant increase after training intervention was found (154). The main problem making the interpretation of these results difficult is the different handling of the samples and the usage of different analysing kits with different sensitivity and detection ranges as well as the different training interventions, the frequency of the training as well as the duration of the intervention. Though studies in healthy controls indicated that the modality of the training as well as the intensity and duration are crucial for the positive effects of training on increasing BDNF levels (155–162), a study in pwMS found no difference of the immediate BDNF increases after continuous moderate or high-intensity interval aerobic training (163). However, aerobic exercise seems to be one of the most potent training forms regarding the effect on neurotrophic factors (164), which was confirmed for BDNF by a recent meta-analysis (154). While studies evaluating the effect of exercise on the expression of neurotrophic factors and the link to neuroprotection in humans have not been conclusive so far (154), animal models already brought strong evidence for exercise-induced increase of BDNF (138, 139, 165–168). Moreover, effects of physical training on cognitive functions mediated by BDNF were analysed in several studies. Hakansson and colleagues found a positive effect on working memory function and peripheral BDNF levels after physical activity (88). Loprinzi and colleagues furthermore reviewed studies focusing on exercise effects on memory and the role of BDNF (73). Most studies found increasing serum BDNF levels after exercise, with 8 studies also blocking BDNF pharmacologically to evaluate exercise effects on memory. When BDNF was blocked, no effects of exercise on memory could be found. Without blocking BDNF, almost half of the studies found a positive effect of exercise on memory (73).

Interestingly, only a few studies assessed the association of training with other characteristics of disease activity as MRI parameters. Savšek and colleagues found protective effects of 12 week aerobic training on atrophy of some substructures as well as a decreasing active lesion volume and count, but without effect on total brain volume, grey matter atrophy, and T2 lesion volume and count as well as cortical lesion count, accompanied with increases of BDNF serum levels in the exercise group (152).

All in all, there is emerging evidence that physical activity increases serum BDNF levels with positive effects on cognition and MRI characteristics. However, the endurance of these increases as well as the effects on physical disability remains subject of further studies. Table 6 provides an overview of the studies assessing the relationship between BDNF and training.

**Table 6:** Overview of the above-mentioned studies investigating the relationship between BDNF and training.

Study	Participants	Parameters	Results
Abbaspoor et al. (142)	20 female patients with MS	Combined functional training or control group for 8 weeks, 3 days per week; measurement of BDNF and IGF1	No difference for BDNF, IGF1 increased in training group
Banitalebi et al. (148)	94 female pwMS	12 weeks (3sessions/week) supervised multimodal exercise program	Increase of BDNF, NT3, NT4/5 levels
Begliuomini et al. (132)	34 male healthy controls	BDNF serum levels (ELISA) every 4 h, 3 times per month	Circadian variations of BDNF with cortisol
Birken et al. (143)	42 patients with progressive MS	9 weeks endurance exercise training; BDNF, Irisin and Il-6 measurement	Increase of BDNF 30 min after bicycling; no changes after 22 sessions of training and no prolonged effects on Irisin and Il-6
Diechmann et al. (154)	Metaanalysis (337 studies, 14 RCTs)	Effect of exercise on neurotrophin levels	9 studies found between group differences for BDNF and 6 out of 12 studies favouring exercise
Dinoff et al. (157)	Metaanalysis (29 studies)	BDNF before and after exercise interventions for at least 2 weeks	BDNF higher after intervention; significant in aerobic, but not resistance training
Dinoff et al. (156)	Metaanalysis (55 studies)	Effect of single-session training on BDNF levels	Single-session training increased BDNF levels in healthy controlsLonger duration of exercises was associated with greater increases of BDNF in males but not in females with higher increases in plasma than in serum
Eftekhari und Etemadifar (149)	30 female pwMS	Pilates 3 times per week for 8 weeks vs control group; BDNF and Il-10 measurement	No changes in Il-10, but increase of BDNF in exercise group
Ferris et al. (158)	15 healthy controls	2 days 30-min endurance training on bike	No significant change in BDNF correlation of BDNF with lactate cognition improved after all exercise conditions, but did not correlate with BDNF changes
Hakansson et al. (88)	23 healthy controls	35 minutes of aerobic exercice at moderate level, cognitive training through a computerized working memory training program or mindfulness practice using an app	Increase of serum BDNF levels after aerobic training, but not after cognitive training oder mindfulness practicePositive association of serum BDNF levels and working memory function
Jørgensen et al. (141)	30 patients with RRMS	24 weeks of resistance training; measurement of BDNF and S1P levels	No changes within or between groups
Khademosharie et al. (150)	24 pwMS	12 weeks resistance and endurance training, 3 sessions per week	No changes in Il-10, butincrease of BDNF in exercise group
Loprinzi (73)	Metaanalysis (52 studies: 26 animal studies, 15 in humans)	Association of cognition and BDNF	All animal studies showed improvement of memory, 97% increase of BDNF with 8 studies showing BDNF as mediator of the positive association of training and cognition.44% of the studies in humans found a positive association of BDNF and cognitive improvement with 40% of these assuming BDNF as mediator
Mandolesi et al. (166)	Animal study (mice)	Running wheel	Positive effect on axonal damage and loss of myelin associated proteins
Mokhtarzade et al. (151)	63 patients with RRMS	Cycling, 3 times a week for 8 weeks, measurement of s100b, NSE, IL10, TNF-a and neurotrophic factors	BDNF and platelet derived factor increased
Naghibzadeh et al. (146)	26 patients with RRMS	4 groups (aquatic exercise, Swedish massage, aquatic exercise + Swedish massage, control group), 3 sessions per week, 30 min each for 8 weeks, measurement of BDNF 48 h before first and after last sessions	BDNF and NGF increased in aquatic exercise alone as well as in combination with Swedish massage while Il-6 decreased
Okzul et al. (147)	36 pw MS, 18 healthy controls	8 weeks combined exercise training; measurement of SOCS1, SOCS4, BDNF	Increase of BDNF after 8 weeks of exercise training
Reycraft et al. (160)	8 male healthy controls	4 weeks of exercise (moderate intensity continuous training; vigorous-intensity continuous training; sprint interval training; no exercise); BDNF before/ immediately after/ 30 minutes after/90 minutes after exercise	BDNF increase depends on exercise intensity
Savsek et al. (152)	28 pwMS	12 weeks aerobic exercise twice a week, control group; BDNF measurement	Only weak increase of BDNF but with positive effect on brain atrophy
Schmolesky et al. (161)	45 healthy male controls	Six exercise conditions based on different intensities on cycle ergometers; BDNF measurement	Increase of sBDNF by 32%, but without effect of exercise intensity or duration on the amount of BDNF increase
Schulz et al. (144)	28 pwMS (immune-endocrine measurements), 39 patients (coordination measurements)	Aerobic training at 60% of VO2max for 30 min; measurement of cortisol, adrenocortico-relasing-hormone, epinephrine, norepinephrine, IL6, sIL6r, BDNF, NGF	No effects on endocrine and immune parameters or neurotrophic factors, but improvement of Quality of Life and coordinative functions
Souza et al. (167)	Animal study (mice)	strength training, endurance and exercise training	Improvement of clinical symptoms with decrease of proinflammatory cytokines (IFN-γ, IL-17, IL-1β), IL-6, MCP-1, TNF-α, and increase of CD25 and IL-10 levels
Szuhany et al. (162)	Metaanalysis (29 studies)	BDNF measurement after single-session vs. regular session exercise vs. resting levels following a program of regular exercise	Moderate effect of single-session exercise on BDNF with greater effect after regular exercise; significant effect of sex with less increase of BDNF in women
Vaynman et al. (140)	Animal study (rats)	4 groups (sedentary with control injection, exercise with contral injection; sedentary with KN-62 injection, exercise with KN-62 injection), voluntary exercise paradigm with running wheel	CAMKII blocked the exercise-induced increase of BDNF and the transcription activator CREB
Waschbisch et al. (145)	83 patients with RRMS	Groups based on subjective report on physical activity; Measurement of T-/B-/NK-cells, Monocytes, regulatory T-Cells; BDNF, Vitamin D, Spiroergomety and TCD	No association of training with BDNF
Wens et al. (153)	22 pwMS, 19 healthy controls	BDNF measurement after exercise program over 24 weeks (5 times per 2 weeks) or control group	Significant increase of BDNF levels in exercise group
Wrann et al. (137)	Animal study (mice)	30 days of endurance training; measurement of Fndc5 gene expression and BDNF (among others)	Increase of BDNF expression by FNDC5, a muscle protein induced by exercise
Xie et al. (168)	Animal study (mice)	Swimming at moderate to high intensity for 6 weeks	No influence on clinical symptoms but reduced infiltration of inflammatory cells and decreased demyelination of spinal cord, increase of IL-10 and TGF-β, as well as BDNF
Zimmer et al. (163)	60 pwMS	High intensity aerobic exercise for 3 weeks vs. standard exercise programm; BICAMS, BDNF measurement	Improvement of verbal memory, but no increase of BDNF levels

## 9 Discussion and conclusion

With increasingly effective therapeutic options to halt inflammation, mechanisms to reverse preexisting, already accumulated damage are becoming increasingly important in context of neuroinflammatory diseases as MS. We reviewed the current literature regarding BDNF, which is thought to play a central role in remyelination and thus neuroplasticity, neuroprotection, and memory, in different areas of MS.

In terms of cognition, studies assuming a positive impact of BDNF on cognitive functions outweigh studies assuming a detrimental effect, although it remains unclear whether the effect is limited on different cognitive domains or whether BDNF improves cognitive functions on a more global level. In this context, especially longitudinal, prospective studies are needed, which also include other so-called “hidden symptoms” of MS such as depression and fatigue.

With regard to the effect of BDNF on disability, especially the improvement of neurological deficits, there is even less evidence, although a positive influence of BDNF on the deficits is also to be favoured here.

However, most of the studies analysed the effect of BDNF on different clinicoradiological characteristics in patients with relapsing disease course or in mixed cohorts mainly consisting of patients with relapsing disease course, therefore, drawing conclusions on the effects of BDNF in patients with progressive disease is difficult. Still, as it is hypothesized that in patients with progressive disease neuroregenerative mechanisms are failing, one could assume that BDNF mainly plays a role in relapsing disease. To date, it is unclear why these mechanisms fail in patients with progressive disease. An exhaustion of BDNF because of compensatory increased synthesis and release in the early years of the disease could be an explanation but needs to be evaluated in further studies with a larger proportion of patients with progressive disease course compared to patients with relapsing disease course.

Furthermore, studies regarding the Val66Met polymorphism have not conclusively determined whether the SNP is a protective or harmful factor in pwMS, but the majority of studies hypothesize a protective effect through modulation of BDNF secretion and anti-inflammatory effects (60). Interestingly, the polymorphism, detectable in up to 72% of the population and thus in a large proportion of healthy subjects (54), seems to have different effects in healthy subjects and pwMS (1, 100). In conclusion, the pro-inflammatory milieu in which BDNF acts in pwMS seems to be essential for these different effects (1, 92, 100). Increased de-methylation of BDNF in an inflammatory milieu could lead to increased secretion and thus neuroprotection. However, Noiciti and colleagues assumed a detrimental effect of increased BDNF secretion as it may result in depletion of the functional reserves and thus faster accumulation of long-term disability (99).

In the author’s opinion, one of the main problems here is the lack of a standardized method for measurement of BDNF. BDNF can be measured in serum as well as normal and platelet-poor plasma. However, as no correlations between serum and plasma BDNF concentrations could be found (169), it has to be assumed that the material used has a relevant influence on the study results. In plasma, BDNF is mainly stored in platelets, therefore, only low concentrations of free BDNF can be assessed. In serum, BDNF is released from platelets due to coagulation. However, in most studies serum was used for measurement of peripheral BDNF concentrations (170). But besides the different material that can be used for measurement of BDNF levels, different methods have been described (170). Mainly, different techniques using immunoassays were used and validated (171), with most studies using enzyme linked immunosorbent assay (ELISA), and to a lesser extent western blot techniques (172). However, since 2013 a single molecule array (SIMOA) technology being able to detect fluid biomarkers on a single-molecule level (171) has been introduced and is increasingly used in studies.

However, despite the inconclusive results and technical difficulties, the author thinks that there is emerging evidence for a neuroprotective and neuroregenerative effect of BDNF in MS, as studies reporting a protective effect outweighed studies assuming detrimental effects of BDNF. BDNF could therefore be a promising biomarker in the field of MS. Still, there is an urgent need for longitudinal prospective studies to improve our understanding of the effects of BDNF in the CNS, especially in context of MS.

Most studies have already shown that BDNF concentrations increase during relapse, although the extent of the increase and thus presumably the neuroregenerative capacity and corresponding recovery from relapses appears to vary between individuals. In consequence, patients with insufficient neuroregenerative capacity could be identified at an early stage and primarily be put on high efficacy therapies. However, understanding which pwMS are at risk of incomplete recovery from relapses by measuring BDNF levels and analysing the association with different clinicoradiological characteristics would significantly improve the clinical management of MS patients.

It has already been shown for glatiramer acetate or IFN-β that BDNF concentrations increase after initiation of these immunotherapies, for glatiramer acetate possibly due to a switch from pro-inflammatory T_H1_ to anti-inflammatory T_H2_ cells. Therefore, further studies comparing the effect of baseline therapies vs. high efficacy therapies on BDNF concentrations are needed. The role of BDNF as a potential therapeutic agent to improve pre-existing neurological deficits should also be analysed in further studies. Nagahara and Tuszynski already reviewed the use of BDNF in different neurological diseases as Alzheimer’s and Parkinson disease as well as spinal cord injuries (173). However, to our best knowledge, similar studies in MS are lacking. Still, as positive results in studies evaluating the effect of BDNF therapy on spinal cord injuries promoting axonal regrowth were found, BDNF could also be a promising approach in multiple sclerosis reversing the demyelination in inflammatory CNS lesions.

## Author contributions

MM: Writing – original draft, Writing – review & editing.

## Glossary

AMP: adenosine monophosphate
AMPA: α-amino-3-hydroxy-5-methyl-4-isoxazolepropionic acid
BDNF: brain derived neurotrophic factor
CaMK: Ca^2+^-calmodulin-dependent protein kinases
CNS: central nervous system
CREB: cAMP response element-binding protein
CSF: cerebrospinal fluid
EAE: experimental autoimmune encephalomyelitis
EDSS: expanded disability status scale
ERK: extracellular-signal regulated kinases
GM: grey matter
HSCT: hematopoietic stem cell transplantation
IFN-β: interferon-β
IP3: inositol triphosphate
MAPK: mitogen-activated protein kinase
MS: multiple sclerosis
mRNA: messenger ribonucleic acid
MTR: magnetization transfer ratio
NAGM: normal appearing grey matter
NAWM: normal appearing white matter
NF-ĸB: nuclear factor ĸB
NMDA: N-methyl-D-aspartate
nt-p75: neurotrophin receptor p75
OPC: oligodendrocyte progenitor cell
PBMC: peripheral blood mononuclear cells
PKC: protein kinase C
PGC-1α: proliferator-activated receptor gamma coactivator 1-alpha
PLC-γ: phospholipase C gamma
PI3-K: phosphatidylinositol-3 kinase
pwMS: patients with MS
RRMS: relapsing remitting MS
SNP: single nucleotide polymorphism
SPMS: secondary progressive MS
TH-cells: T-helper cells
TNF: tumor necrosis factor
TNFR: tumor necrosis factor receptor
Trĸβ: tropomyosin-related kinase β
WM: white matter
